# Spontaneous T Cell Proliferation: A Physiologic Process to Create and Maintain Homeostatic Balance and Diversity of the Immune System

**DOI:** 10.3389/fimmu.2018.00547

**Published:** 2018-03-19

**Authors:** Booki Min

**Affiliations:** ^1^Department of Immunology, Lerner Research Institute, Cleveland Clinic Foundation, Cleveland, OH, United States

**Keywords:** lymphocytes, homeostasis, T cells, proliferation, lymphopenia

## Abstract

Naive T lymphocytes undergo heterogeneous proliferative responses when introduced into lymphopenic hosts, referred to as “homeostatic proliferation” and “spontaneous proliferation.” Spontaneous proliferation is a unique process through which the immune system generates memory phenotype cells with increasing T cell receptors repertoire complexity. Here, the mechanisms that initiate and control spontaneous proliferation are discussed.

The immune system is constantly exposed to foreign antigens derived from microbes that are either harmless or pathogenic and to self-antigens that are either normal or transformed. The fundamental goal of such immunological practices is to achieve two outcomes: to eliminate harmful pathogens and transformed cells while to preserve harmless commensal microbes and normal cells.

T cells are a trustworthy fighter that identifies and eliminates invading pathogens, yet a potential traitor capable of attacking self-tissues and causing autoimmunity. The immune system has thus evolved several lines of checkpoints that ensure safety and loyalty of developing lymphocytes. Thymocytes that express useless or harmful antigen receptors are eliminated by apoptotic cell death, while those expressing self-MHC restricted antigen receptors with moderate reactivity to self-antigens are allowed to mature. T cells that survive the selection processes and leave the thymus to express the T cell receptors (TCR) displaying a measurable, yet weak reactivity against self-antigens that is not strong enough to cause autoimmunity, yet sufficient enough to maintain both survivability and reactivity to the subsequent antigen encounter (1, 2). Those T cells selected by relatively stronger interaction toward self-antigens may further develop into T cells with regulatory functions (3–5). In the periphery, T cells constantly receive a “tonic” signal from interacting with MHC^+^ antigen presenting cells (APCs), resulting in a partial phosphorylation of the ζ chain of the TCR complexes (2). This is critical to maintain proper reactivity and survival of T cells, although the impact on the latter remains controversial (2, 6). Since developing thymocytes are selected based on “self-reactivity,” the immune system develops an additional measure to prevent unnecessary T cell activation in response to self-antigens in the periphery, including anergy and regulatory T cells. The failure in such regulatory mechanisms results in uncontrolled autoimmune inflammatory responses. Thus, keeping the regulation under control is a matter of utmost importance.

The number and composition of T cells in the periphery is tightly controlled at a relatively constant level throughout life, suggesting the existence of a homeostatic mechanism(s). Alterations in the homeostasis trigger series of compensatory mechanisms that reinstate homeostatic equilibrium. For example, viral infection causes clonal expansion of antigen-specific CD8 T cells, during which peripheral CD8 T cells massively expand and up to ~90% of the total CD8 T cells may become antigen specific in case of lymphocytic choriomeningitis virus infection (7). Once the infection is cleared, homeostasis returns to the normal level as the majority of the expanded effector cells are eliminated, leaving newly generated virus-specific memory T cells. Lymphopenia incurred from normal physiologic processes that occur during neonatal period, from pathogenic conditions, such as viral infection, or from therapeutic interventions triggers T cell proliferation that restores the T cell deficiency (8–10). Therefore, T cell homeostasis is a key process that requires a precise balance between proliferation and apoptosis. A plethora of evidence indicates that dysregulation in T cell homeostasis can lead to inflammatory disorders, including autoimmune diseases, HIV-associated immune reconstitution inflammatory syndrome, Omenn syndrome, bare lymphocyte syndrome, and others (11–14).

## T Cell Proliferation in Lymphopenic Settings, A Model to Study T Cell Homeostasis

For the past decades, examining T cell proliferation under lymphopenic settings has been the primary *in vivo* model system to investigate mechanisms controlling T cell homeostasis and immunopathology associated with homeostatic imbalance. Pioneering studies from the Chen and Bevan groups demonstrated that naive CD8 T cells transferred into irradiated or Rag^−/−^ recipients undergo proliferative responses without “cognate antigen” stimulation and acquire a memory-like phenotype (15, 16). The proliferative potential of such responses was once estimated that one T cell has a potential to generate 10^15^ progenies during the process (17).

## Spontaneous Proliferation vs. Homeostatic Proliferation

While earlier studies interchangeably utilized mild and severe lymphopenic models to investigate proliferative T cell responses inclusively called homeostatic proliferation (or lymphopenia-induced proliferation), subsequent studies uncovered that T cell proliferation within lymphopenic settings is highly heterogeneous. We reported that there are at least two mechanistically distinct proliferation modes referred to as spontaneous proliferation and homeostatic proliferation (18). Spontaneous proliferation is a robust proliferation found in “severe” lymphopenic hosts, including mice with mutation in genes involved in lymphocyte generation. Spontaneously proliferating cells divide more than a cell division per day even in the absence of homeostatic cytokines (18, 19). In case of CD4 T cells, the requirement for spontaneous proliferation is rather unique, because MHC II molecules expressed on CD11c^+^ dendritic cells (DCs), but not on B cells are required for proliferation (20). The requirement for naive CD8 T cell spontaneous proliferation is less rigorous, and either MHC I or MHC II expressed on DCs or B cells are sufficient to induce proliferation (20). Additional important feature for spontaneous proliferation is that the proliferating cells turn into phenotypically different populations. They rapidly differentiate into memory phenotype cells, acquiring memory/effector cell markers and an ability to produce inflammatory cytokines upon stimulation (18). Unlike T cells activated by cognate antigen, however, spontaneously proliferating T cells do not express early activation markers (CD69 and CD25), although CD44 upregulation and CD62L downregulation still occurs, allowing them to preferentially migrate into non-lymphoid tissues as antigen-stimulated effector/memory T cells do. Homeostatic proliferation is a slow response that occurs within “mild” lymphopenic conditions following sublethal irradiation or T cell ablation in the presence of functionally intact thymus (18, 21). Homeostatically proliferating CD4 T cells undergo a cell division every 3–4 days, although CD8 T cell proliferation is considerably faster than that of CD4 T cells (18). TCR interaction with MHC:peptide complexes is instrumental for the responses as blocking the interaction inhibit proliferation (22, 23). However, TCR engagement alone is not sufficient for proliferation. Treatment with neutralizing antibodies against homeostatic cytokine, namely IL-7, significantly inhibits homeostatic proliferation of T cells (18). Therefore, signals generated from the TCR and the cytokine receptors must be incorporated to trigger proliferation. The nature of antigens involved in homeostatic proliferation remains unclear. However, it is likely low affinity self-antigens because homeostatic proliferation is not impaired in germ-free lymphopenic recipients (19).

## Quantitative and Qualitative Signaling Models

**Figure 1:**
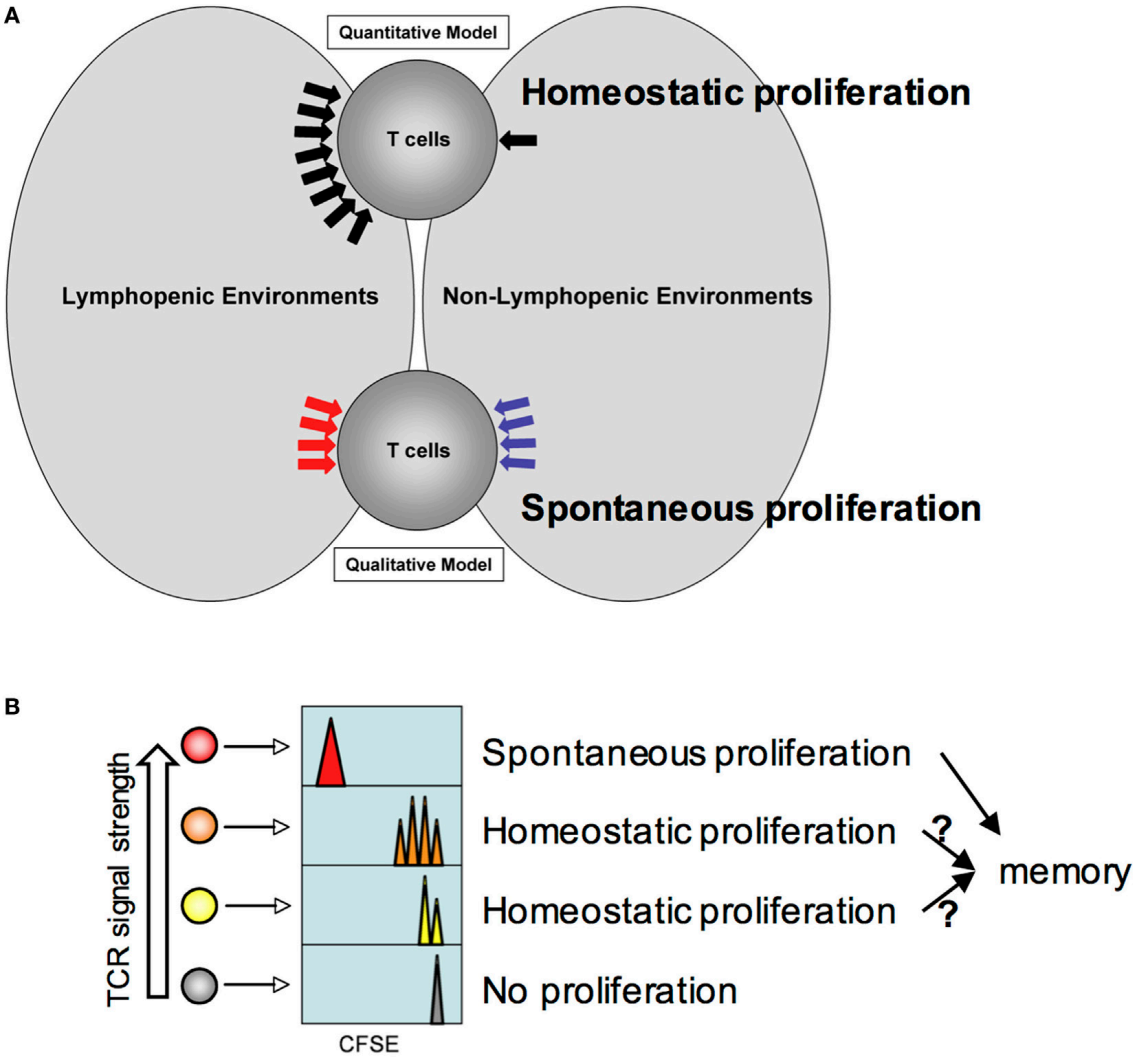
Model for homeostatic and spontaneous proliferation. **(A)** Quantitative and qualitative signaling model. The model depicts potential signaling mechanisms during homeostatic and spontaneous proliferation. Homeostatic proliferation is triggered by excessive soluble resources available under lymphopenic environments. By contrast, spontaneous proliferation is triggered by different types of signaling mechanism only available under lymphopenic conditions. **(B)** Relative T cell receptors signal strength against endogenous peptide:MHC complexes determines which T cells undergo homeostatic and spontaneous proliferation. The higher the strength is, the more likely the T cells would undergo spontaneous proliferation. If the strength is below threshold, cytokine availability controls homeostatic proliferation. T cells would remain undivided.

To account for the distinct nature and underlying mechanisms underlying homeostatic and spontaneous proliferation we propose the quantitative and qualitative signaling models (Figure 1A). The quantitative signaling model for homeostatic proliferation postulates that the relative amount of available resources determines the mode of T cell proliferation. The level of serum IL-7 is found significantly higher in lymphopenic hosts (24, 25). In fact, IL-7 production by stromal cells appears to be controlled as a part of homeostatic mechanism (24), through which peripheral T cell survival, proliferation, and apoptosis are balanced. In addition, the relative abundance of lymphocytes in the periphery may further determine the competition. In Rag^−/−^ recipients, a low competition (i.e., more availability) for IL-7 promotes cell survival by enhanced expression of anti-apoptotic factors and cell proliferation by degrading cell cycle inhibitor p27 (26). Homeostatic proliferation is a dominant response in these environments. However, the level of IL-7 available is likely lower in TCRβ^−/−^ or TCR transgenic mouse recipient due to competing endogenous B cells or transgenic T cells. Due to competition for IL-7, homeostatic proliferation is not typically observed in these recipients (18, 27). However, provision of exogenous IL-7 induces homeostatic proliferation in such conditions, supporting the importance of IL-7 during homeostatic proliferation. Moreover, the extent of proliferation is similar to that observed in Rag^−/−^ or sublethally irradiated recipients and is proportional to the amount of given IL-7 (18). T cells transferred into lympho-replete wild type recipients remain undivided, and providing exogenous IL-7 is sufficient to trigger homeostatic proliferation of the transferred cells in lymphocyte-sufficient environments (18).

The qualitative signaling model for spontaneous proliferation postulates that the nature of signals that T cells receive is fundamentally different from those that T cells receive within lymphocyte-sufficient conditions (Figure 1A). IL-7 or other homeostatic cytokines play little or no role in this response. Instead, antigens originated from commensal microbes appear essential in inducing spontaneous proliferation as proliferation is considerably reduced in germ-free lymphopenic animals (19). However, it is worth noting that a measurable spontaneous proliferation is still observed in germ-free lymphopenic hosts, suggesting a contribution of food or self-antigens. Given the fast proliferation dynamics and full differentiation, it is likely that the signaling cascade may be analogous to that of cognate antigen-induced T cell activation.

The molecular basis underlying spontaneous and homeostatic proliferation has been tested using various gene knockout animals. Zamoyska and colleagues reported using the p56^lck^-deficient system that sustained lck expression is required for the proliferation of CD4 and CD8 T cells (28). Lck deficiency in transferred T cells impairs homeostatic and possibly spontaneous proliferation of both CD4 and CD8 T cells (28). However, the study was carried out over 3–6 weeks post transfer. The study is thus not suitable to determine the exact requirement of lck during spontaneous proliferation. Shen et al. examined the importance of the LAT (linker for activation of T cells) during T cell proliferation in lymphopenia (29). They also measured LAT^−/−^ T cell proliferation in T cell-deficient LAT^−/−^ recipients, where T cell development is blocked. LAT deficiency greatly impairs T cell proliferation, suggesting that LAT expression may be necessary for spontaneous proliferation (29). Gascoigne and colleagues examined the role of different protein kinase C isoforms and found that PKCη, but not PKCθ plays a key role in regulating homeostatic proliferation of CD8 T cells in sublethally irradiated recipients (30). Signaling pathways that occur during spontaneous proliferation remain to be investigated.

Does each T cell express equal potential to undergo homeostatic and/or spontaneous responses and are they stochastically selected? Or alternatively, are they predetermined for either but not for both responses? The strength of “tonic” signals may be a key factor determining which cells are selected to respond to homeostatic or endogenous cues. Peripheral mature T cells are a highly heterogeneous population such that each T cell clone expresses the antigen receptors with spectral affinity against self (and/or commensal) antigens. The heterogeneity (i.e., the strength of such interaction) can be reflected by the level of surface expression of CD5, a negative regulator of TCR signaling (31). An earlier study where CD5^hi^ or CD5^lo^ naïve T cells were purified and transferred into mild lymphopenic recipients showed more extensive homeostatic proliferation of CD5^hi^ T cells in sublethally irradiated recipients (32). Therefore, T cells expressing higher affinity antigen receptors are likely to participate in spontaneous proliferation, although this hypothesis needs to be tested. The proliferating cells turn into memory phenotype cells and play a central role in regulating spontaneous proliferation of naive T cells (see below). On the other hand, T cells expressing low or moderate affinity receptors are expected to undergo homeostatic proliferation or remained undivided (Figure 1B). Interestingly, a correlation between slow cell division and CD44 upregulation was observed during homeostatic proliferation (18). These cells may eventually differentiate, if allowed, into memory phenotype cells analogous to those generated from spontaneous mechanism and participate in enhancing the memory cell repertoire complexity (see below). Alternatively, there may exist a threshold that allows proliferating cells to become functional memory cells (33). Marginal upregulation of CD44 expression in homeostatically proliferating cells may become transient and reversible as previously observed (15). Foxp3^+^ regulatory T cells are thought to be selected from developing T cells recognizing self-antigens with a higher affinity (34–36). Indeed, Tregs or CD44^hi^ memory phenotype CD4 T cells are known to undergo more extensive spontaneous proliferation even within lymphocyte-sufficient environments (37, 38).

## Principles for Spontaneous Proliferation

**Figure 2:**
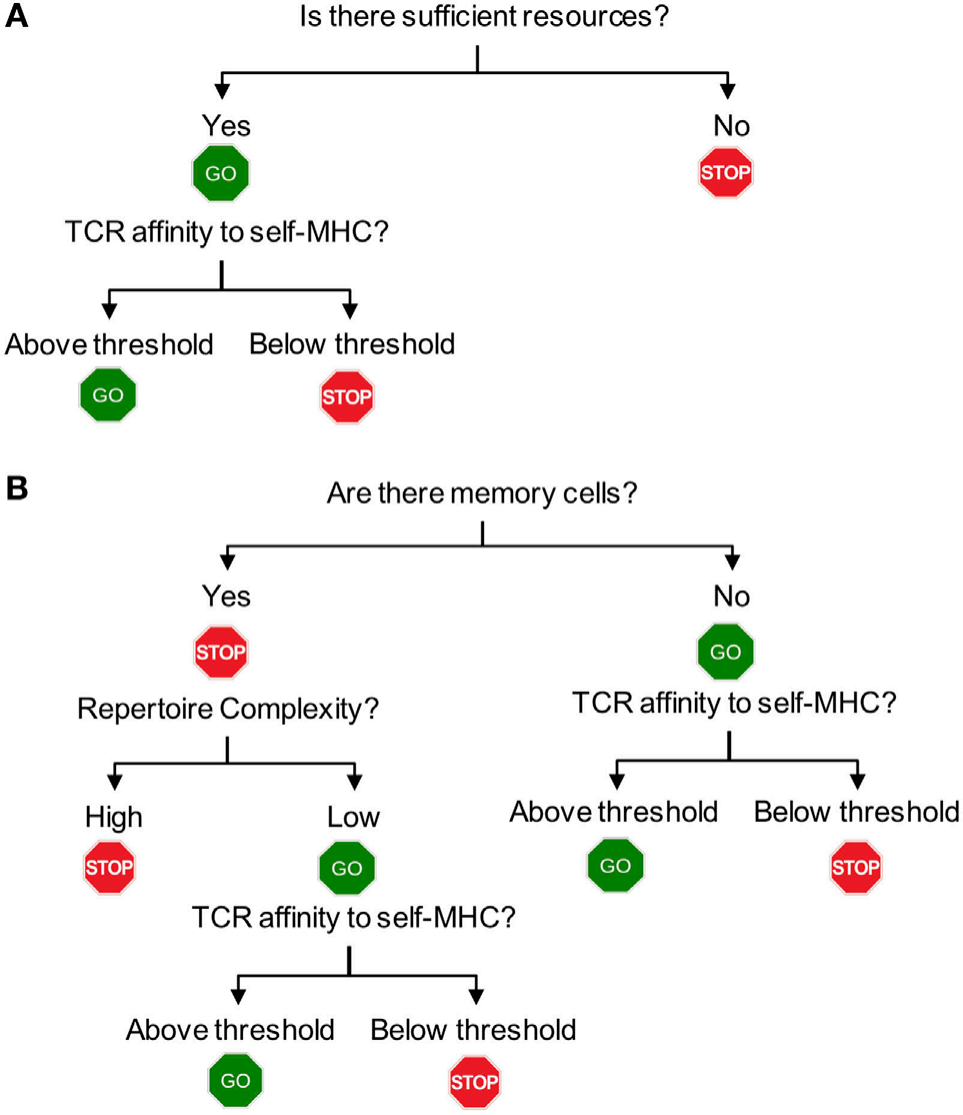
Flowchart for homeostatic and spontaneous proliferation. **(A)** Homeostatic proliferation. The first step for T cells to check is whether there is sufficient homeostatic factor available. Once the source is available, then the relative affinity of each T cells expresses against MHC-self-antigens (or exogenous antigen as well) will determine proliferation. **(B)** Spontaneous proliferation. Unlike homeostatic proliferation, the first step deciding spontaneous proliferation is whether there is a memory population in the periphery. The lack of any memory population triggers a full blown spontaneous proliferation during which T cells with higher affinity to MHC-self-antigens (and commensal antigens) are induced for proliferation. If there is a population of pre-existing memory cells, the complexity of T cell receptors (TCR) repertoire will then become the next step determining proliferation. Only incomplete repertoire complexity of memory cells will allow spontaneous proliferation to occur. The final decision will depend upon the TCR that each T cell expresses. Only if the TCR strength toward endogenous peptides (self or commensal) is above threshold, they will be allowed to undergo proliferation.

Examining spontaneous proliferation has uncovered several unique features that are distinct from those operated during homeostatic proliferation (Figure 2). *First, spontaneous proliferation is triggered by the lack of memory T cells not by the total T cell numbers*. The earlier notion accounting for T cell proliferation within lymphopenic environments was that T cells “sense” the existence of neighboring T cells. Homeostatic proliferation competing for soluble resources is properly explained by the notion. However, the finding that spontaneous proliferation is comparably induced when T cells are transferred “into” TCR transgenic recipients, where wild type level peripheral T cells with monoclonal TCR repertoire and primarily naive phenotype exist strongly suggests that the total number of peripheral T cells is not a factor for proliferation. In fact, the absence of memory T cells in this condition is the signal triggering proliferation. *Second, the clonality of peripheral T cells determines spontaneous proliferation*. However, it was further shown that the presence of memory cells itself cannot be a sole factor determining spontaneous proliferation (Figure 2B). TCR transgenic T cells undergo spontaneous proliferation when transferred to TCR transgenic hosts with a different clonotype, while remain undivided when transferred to the hosts with the same clonotype, demonstrating the importance of TCR clonality (39). One may also postulate that memory T cells may better compete for homeostatic resources, thereby efficiently inhibiting spontaneous proliferation. Then, it is predicted that TCR transgenic recipients with abundant memory cells (of the same specificity) would be sufficient to control the proliferation. Immunizing TCR transgenic mice with cognate antigens by which large proportions of peripheral T cells become effector/memory cells is unable to inhibit spontaneous proliferation of polyclonal naïve T cells (40), suggesting that the clonality of memory T cells is crucial. *Third, the repertoire complexity of memory T cells controls spontaneous proliferation*. Varying numbers of T cells transferred into immunodeficient mice form memory phenotype cells with different repertoire complexity. Importantly, the total number of memory phenotype cells generated from different T cell inoculums’ remain relatively similar, suggesting that there appears to be a homeostatic mechanism that maintains the size of memory T cells (40). When the second cohorts of naïve T cells are introduced into these recipients, the extent of spontaneous proliferation of newly transferred cells is directly determined by the repertoire diversity of the pre-existing memory cells (40). When pre-existing memory T cell repertoire complexity is low, the extent of new spontaneous proliferation from the second naïve T cells is greater. By contrast, pre-existing memory T cells with higher repertoire complexity efficiently limit spontaneous proliferation of new naïve T cells. Direct comparison of the TCR clonality between the pre-existing and newly formed memory phenotype cells demonstrates that their TCR clonality is mostly non-overlapping (40). Therefore, it is concluded that a “hole” in the memory T cell repertoire allows naive T cells that are capable of recognizing antigens occupying the hole to undergo spontaneous proliferation and to differentiate into memory cells (41). As the result, the “hole” would be filled up by these newly formed memory phenotype cells, increasing the overall repertoire complexity and becoming homeostatically “stable.”

## Regulation of Spontaneous Proliferation

The fact that spontaneous proliferation is diminished in germ-free immunodeficient hosts strongly suggests that antigens derived from commensal organisms are the primary source supporting proliferation and differentiation. However, the very similar commensal antigens are also likely presented in a lympho-replete condition possibly in a tolerogenic fashion. *Why do commensal antigens fail to trigger spontaneous proliferation in this case?* We already discussed that the presence of memory phenotype cells with higher repertoire complexity controls the induction of spontaneous proliferation. *Then, how does it operate?*

**Figure 3:**
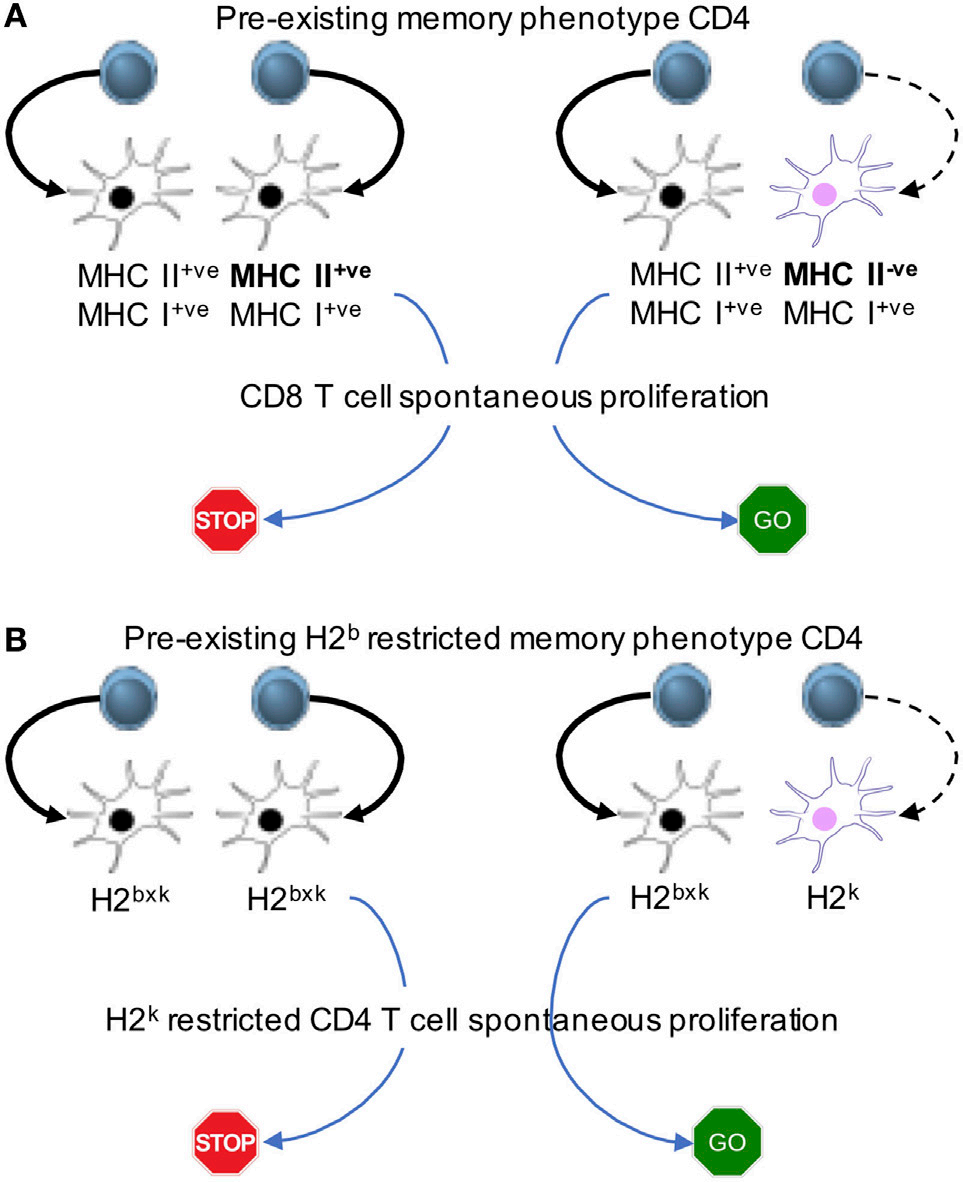
Memory cell-induced regulation of spontaneous proliferation operates *via* antigen presenting cells (APCs). Mixed bone marrow chimeras in which different APC populations expressing MHC I and II or MHC I alone **(A)** or expressing different MHC II haplotypes **(B)** are created. The first cohort of CD4 T cells is transferred to generate “pre-existing” memory phenotype cells. The second cohort of naive CD8 **(A)** or naive CD4 **(B)** cells is transferred into the recipients. The model system uncovers that the interaction between APCs and pre-existing memory phenotype CD4 T cells is essential to limit the proliferation of new naive T cells.

Reconstituting immunodeficient mice with two cohorts of T cells at different time points gives us a system, where one could examine the underlying cellular mechanisms. Pre-existing memory phenotype CD4 T cells limit spontaneous proliferation of both naive CD4 and CD8 T cells (23). The hypothesis is that the memory T cells inhibit naive T cell proliferation *via* altering APC functions. By reconstituting mice with a mixture of TCRβ^−/−^ bone marrow progenitors that express MHC II or not, we create a mouse model in which half of the bone marrow-derived APCs express MHC II (and the other half of the APCs are derived from MHC II^−/−^ bone marrow progenitors), while equally expressing MHC I molecules. Transferring CD4 T cells will trigger spontaneous proliferation, which will differentiate into memory phenotype cells. The pre-existing memory phenotype CD4 T cells would be interacting with MHC II^+^ APCs, while MHC II^−/−^ APCs would remain “untouched” by those memory cells. Freshly transferred naive CD8 T cells undergo spontaneous proliferation even in the presence of functionally competent memory phenotype CD4 T cells only if the half of the APCs does not express MHC II (Figure 3A). We also examine if memory CD4 T cells inhibit naïve CD4 T cell spontaneous proliferation *via* similar mechanism. Bone marrow chimeras harboring different haplotype MHC II molecules (for example, haplotype H2*b* and H2*k*) are created (Figure 3B). Memory CD4 T cells restricted to the H2*b* haplotype are able to limit spontaneous proliferation of naïve CD4 T cells, restricted to the H2*k* haplotype only when all APCs do express both haplotypes. By contrast, spontaneous proliferation is strongly induced when some APCs express the H2*k*, but not the H2*b* (Figure 3B). T cells may down-modulate peptide-MHC complexes on APCs, inhibiting T cell responses to the same peptide-MHC complexes (42). We found that memory T cell interaction with especially DCs induce the production of IL-27 from CD8^+^ DC subsets, and that IL-27 plays a central role in regulating spontaneous proliferation of naïve CD4 and CD8 T cells, because IL-27R^−/−^ naïve T cell spontaneous proliferation is not affected by the presence of memory phenotype CD4 cells with complex repertoire diversity and of fully competent APCs (23).

## Spontaneous Proliferation in a Physiologic Setting

Does spontaneous proliferation occur in a physiologic setting? We previously reported that naïve T cells transferred into wild type newborn mice undergo spontaneous proliferation (10). During postnatal life, the peripheral lymphoid tissues are rapidly occupied by recent thymic emigrants (43). Those early emigrant T cells are highly proliferating cells as determined by BrdU incorporation and differentiate into memory phenotype cells (44). In fact, delaying the T cell transfer at different postnatal life greatly diminishes spontaneous proliferation of the T cells, suggesting a competition from endogenously generated T cells (10). What is the immunological significance of these spontaneously generated memory phenotype cells? Recent report from Kawabe et al. elegantly demonstrated that these memory phenotype cells acquire T-bet transcription factor expression in response to endogenously supplied IL-12 (38). Most importantly, this study also showed that these IFNγ-producing memory phenotype cells provide a nonspecific host resistance against Toxoplasma infection, enhancing the adaptive immune responses (38). These cells resemble “virtual memory” CD8 T cells expressing foreign antigen-specific memory phenotype in unimmunized animals that are generated by homeostatic mechanisms (45, 46).

## Outstanding Questions

In conclusion, I would argue that spontaneous proliferation is a key homeostatic process by which endogenous memory phenotype cells are generated and their repertoire complexity increases. There are several key questions worth re-visiting. First, we know very little on the molecular pathways that activate naive T cells to support their differentiation into memory phenotype cells. Although there have been earlier studies examining signaling mediators involved in T cell proliferation within lymphopenic environments, the precise signaling cascade underlying spontaneous proliferation remains poorly understood. What are the key kinases activated during spontaneous proliferation and are they different from cognate antigen-induced activation or homeostatic proliferation? Second, little is known about the antigens that trigger spontaneous proliferation. Are there specific commensal microbe antigens supporting proliferation? Which self-antigens are capable of inducing spontaneous proliferation? Is TCR strength against self-antigens reflected by CD5 expression playing a role in inducing spontaneous proliferation? Last, do Foxp3^+^ regulatory T cells undergo spontaneous proliferation and is the repertoire complexity of regulatory T cells similarly completed through spontaneous mechanisms seen in conventional T cells? Given the self- or commensal-antigen-driven nature of spontaneous proliferation, it is likely that autoimmunity or inflammatory responses in the intestinal tissues may be induced by dysregulated spontaneous proliferation.

## Personal Note

From June 2000 to October 2014, I have had the privilege of working in the Laboratory of Immunology of the NIAID under the guidance of Bill. One day after I joined the lab, he asked me a question. “Will T cells divide in newborn mice ?” Since my Ph.D. project was about neonatal tolerance, his question sounded something testable (and most importantly doable). So, I did the experiment, which changed my interest in Immunology since then. I became fascinated by the concept of lymphocyte homeostasis, which I would still call the main backbone of my current research program. The fact that I was the only fellow in the lab who does not work on IL-4 or on Th2 differentiation did not bother me at all. I still remember how much I had enjoyed doing experiments day and night, preparing and analyzing data for the weekly meeting, discussing the data with him, and planning the next experiments. Nurturing and supportive environment at the Laboratory of Immunology, other faculty members I often interacted with (Ethan Shevach, Ron Germain, Alan Sher, etc.) and colleagues (Jeff Zhu, Lily Guo, Hidehiro Yamane, Javier Cote-Sierra, Nobuki Hayashi, Gilles Foucras, Graham Le Gros, Zami Ben-Sasson, Cyndy Watson, Jane Hu-Li, Irena Stefanova, Dragana Jankovic, and Zvi Grossman), and Bill. It was the perfect combination of all. Even after I had left the lab to run my own laboratory, he continued to inspire and teach me. Thank you, Bill and you are immensely missed.

## Author Contributions

The author confirms being the sole contributor of this work and approved it for publication.

## Conflict of Interest Statement

The author declares that the research was conducted in the absence of any commercial or financial relationships that could be construed as a potential conflict of interest.
